# Complex Prospective Memory in Adults with Attention Deficit Hyperactivity Disorder

**DOI:** 10.1371/journal.pone.0058338

**Published:** 2013-03-06

**Authors:** Anselm B. M. Fuermaier, Lara Tucha, Janneke Koerts, Steffen Aschenbrenner, Celina Westermann, Matthias Weisbrod, Klaus W. Lange, Oliver Tucha

**Affiliations:** 1 Department of Clinical and Developmental Neuropsychology, University of Groningen, Groningen, The Netherlands; 2 Department of Clinical Psychology and Neuropsychology, SRH Clinic Karlsbad-Langensteinbach, Karlsbad-Langensteinbach, Germany; 3 Department of Psychiatry and Psychotherapy, SRH Clinic Karlsbad-Langensteinbach, Karlsbad-Langensteinbach, Germany; 4 Section for Experimental Psychopathology, Centre for Psychosocial Medicine, University of Heidelberg, Heidelberg, Germany; 5 Department of Experimental Psychology, University of Regensburg, Regensburg, Germany; Hangzhou Normal University, China

## Abstract

**Objectives:**

Attention deficit hyperactivity disorder (ADHD) in adults has been associated with disturbances of attention and executive functions. Furthermore, impairments of verbal and figural retrospective memory were reported. However, little is known about the effects of ADHD on prospective memory, the execution of delayed intentions in the future.

**Methods:**

The present study compared the performance of 45 adult patients with ADHD not treated with stimulant medication with the performance of 45 matched healthy individuals on a paradigm of complex prospective memory which measured task planning, plan recall, self-initiation and execution. Furthermore, the contribution of other cognitive functions to prospective memory functioning was assessed, including measures of attention, executive functions and memory.

**Results:**

A large-scale impairment could be observed in task planning abilities in patients with ADHD. Only negligible to small effects were found for plan recall, self-initiation and execution. Inhibition was identified to contribute significantly to performance on task planning.

**Conclusions:**

The present findings suggest that four cognitive components contribute to the performance of prospective memory. Impairments of prospective memory mainly emerged from deficient planning abilities in adults with ADHD. Implications on behavioral based intervention strategies are discussed.

## Introduction

Behavioral characteristics of attention deficit hyperactivity disorder (ADHD) are symptoms of inattention, hyperactivity and impulsivity and have been studied for many years [1], [2]. Whereas overt behavioral disturbances (e.g. symptoms of hyperactivity) are more strongly associated with childhood ADHD, cognitive inefficiency is reported to be more pronounced in the adult form of the disorder [3]. Therefore, assessment of cognitive functions is of particular importance in adults with ADHD. Neuropsychological research in ADHD primarily focused on aspects of attention and executive functions [3], [4]. With regard to attention, deficits of vigilance, selective attention, distractibility, divided attention and flexibility have been observed [5]–[8]. In the domain of executive functions, impairments of working memory, inhibition, set shifting and planning have repeatedly been found [9]–[12].

More recently, theoretical considerations and empirical research have suggested that impairments associated with executive functions may lead to memory problems in patients with ADHD. For example, meta-analyses on neuropsychological test performance of adults with ADHD revealed medium effect sizes for verbal memory disturbances and up to small effects sizes for figural memory disturbances [13], [14]. While the majority of studies explored *retrospective memory* functions, *prospective memory* functions have largely been neglected. *Prospective memory* is a term which refers to the memory to perform an intended action at a particular point in the future. Therefore, *prospective memory* can also be described as 'the delayed execution of an intended action' [15], [16]. Tasks of prospective memory in daily life involve keeping an appointment or giving a message to a friend or colleague. In the clinical setting, the importance of prospective memory for medication adherence was shown in several studies [17]. The paucity of empirical research on prospective memory in individuals with ADHD appears surprising as prospective memory represents an essential ability for everyday life functioning. The high relevance of prospective memory for everyday life and clinical practice was stressed by Kliegel and Martin [18]. These authors argued that 50% to 80% of all memory problems in daily life were, at least in some point, problems of prospective memory [18]–[20]. This is in accordance with the finding that 40% of all patients of a memory clinic reported prospective memory problems as their main symptoms [18]. Successful completion of tasks measuring prospective memory has been found to be strongly associated with a variety of executive functions such as planning, working memory, switching and inhibition [15], [21]. As ADHD is characterized by executive dysfunction [10], [22]–[24], individuals with ADHD have been assumed to suffer from impairments in prospective memory [25], [26].

To date, there are five studies examining aspects of prospective memory in patients with ADHD. Whereas four studies are focusing on children with ADHD [27]–[30], there is only one study exploring prospective memory in adults with ADHD [31]. Differential effects were found with regard to the performance in time-based and event-based prospective memory [32]. Time-based prospective memory which requires the execution of an intended action at a specific time in the future (e.g. calling a friend at 3 pm), was found to be impaired in both children and adults with ADHD [28], [30], [31]. However, event-based prospective memory which requires the execution of an intended action in response to a specific event (e.g. giving a friend a message during the next meeting), was not affected in children and adults with ADHD [27], [31]. Experiments on time-based and event-based prospective memory are in general limited by the often isolated character of the performance of single actions in the future (e.g. pressing a button at a predefined time or event). The ecological validity of some of these paradigms might be questionable considering that in daily life often multiple tasks need to be integrated in one plan for a later execution. In the attempt to develop a more realistic measure of multiple delayed intentions, Kliegel and colleagues suggested a valuable paradigm of complex prospective memory [33]. The authors differentiated between (I) complex task planning, (II) plan retention, (III) self-initiation of the task performance at a predefined point (event or time) and (IV) task execution. This paradigm allowed to differentiate between several cognitive requirements of prospective memory and has been shown to be sensitive in several populations with cognitive dysfunction, including children with ADHD [29], patients with Parkinson’s disease [34] or individuals suffering from traumatic brain injury [35]. Accordingly, a very recent study on prospective memory in adults with ADHD showed impairments in task planning and plan adherence in task execution [31]. However, plan retrieval and self-initiation of task execution have unfortunately not been examined yet in adults with ADHD.

The aims of the present study were (I) to create a task which is sensitive to measure complex prospective memory in adults with ADHD (according the principles suggested by Kliegel and colleagues [33]), (II) to replicate findings of impaired planning and plan adherence in adults with ADHD as reported by Altgassen [31] (i.e. sample-size calculation), (III) to enlarge our current knowledge about prospective memory in adult ADHD by examining task planning, plan retention, self-initiation and execution in one integrated paradigm and (IV) to find out which cognitive weaknesses affect the complex prospective memory of adults with ADHD. Consequences of the present results, such as implications on the interaction in behavioral based interventions (i.e. cognitive-behavioral therapy) will be discussed.

## Methods

### Participants

A total number of 45 adult patients with ADHD participated in the study. The required sample size was calculated according to the differences as reported by Altgassen [31] between adults with ADHD and healthy participants in task planning (Cohen’s d = 0.64) and plan adherence (Cohen’s d = 1.06). Based on a t-test of independent samples, a power (1−β) of 0.85 and a significance level of 0.05, a sample size of 45 participants per group is required to replicate group differences in task planning and plan adherence with sufficient certainty [36].

All patients were outpatients, recruited from the Department of Psychiatry and Psychotherapy, SRH Group, Karlsbad-Langensteinbach, Germany. The diagnostic assessment was performed by experienced clinicians and involved a clinical psychiatric interview according to DSM-IV criteria for ADHD as devised by Barkley and Murphey [37] including the retrospective diagnosis of an ADHD in childhood (DSM-IV criteria) and current symptoms. Moreover, all participants completed two standardized self-report rating scales designed to quantify current and retrospective ADHD symptoms [38]. Childhood ADHD symptoms were self-rated with the short version of the Wender Utah Rating Scale (WURS-K) including 25 items on a five-point Likert scale [39]. Severity of adulthood ADHD symptoms was self-rated with the ADHD self-report scale [38] consisting of 18 items on a four-point Likert scale corresponding to the diagnostic criteria of DSM-IV [2], [38]. Patients were selected according to age, diagnosis, intellectual functions (IQ), and willingness to participate in the study. Potential patients were excluded (I) if they had clinically significant chronic medical conditions, (II) if they were currently treated with psychostimulants, (III) if there was a history suggestive of ‘psychosis’ (indicating schizophrenia, delusional disorder, depressive disorder with psychotic features or manic episode), (IV) if there was a history of neurological disorder including head injury, (V) if there was a history of substance abuse disorder during the previous two months, (VI) if the initial psychiatric assessment indicated a current major depressive episode or (VII) if estimated verbal IQ was <85. In the diagnostic assessment of the 45 patients with ADHD, 14 patients met DSM-IV criteria for ADHD – predominantly inattentive type (ADHD-I), 1 patient met criteria for ADHD – hyperactive-impulsive type (ADHD-H) and 30 patients met criteria for ADHD – combined type (ADHD-C). Fifteen of the 45 patients with ADHD were diagnosed with one or more comorbid disorders, including mood disorders (n = 11), anxiety disorders (n = 2), personality disorders (n = 2), eating disorders (n = 2) and substance abuse disorder (n = 1). At the day of the assessment, 8 patients were treated with antidepressive medication because of affective disorders.

Furthermore, 45 healthy individuals were assessed. None of the healthy individuals reported to have a history of neurological or psychiatric disease and none were taken any medication known to affect the central nervous system at the day of the assessment. All healthy individuals were recruited from the local community and completed the same self-rated questionnaires for current and retrospective ADHD symptoms prior to the assessment [38]. Intellectual functions of all individuals were measured using the Multiple Choice Vocabulary Test [40]. Patients and healthy individuals did not differ in age (t(88) = –0.79, p = .43), gender (exact same distribution in both groups) and intellectual functions (t(88) = 0.57, p = .57). As expected, healthy individuals scored lower on both questionnaires for ADHD symptoms (t(88) = −14.55, p<.001 for current symptoms; t(88) = −13.64, p<.001 for retrospective symptoms). Characteristics of patients with ADHD and healthy individuals are presented in Table 1.

**Table 1:** Characteristics of participants.

	Patients with ADHD (n = 45)	Healthy individuals (n = 45)
Age (in years)	34.9±10.9	33.1±9.8
Gender (female/male)	23/22	23/22
Intellectual functions (IQ)^a^	100.5±11.4	101.8±10.1
WURS-K^b^	46.3±13.5	12.2±8.8
ADHD – self-report scale	33.8±9.0	9.8±5.6

a Multiple Choice Vocabulary Test (MWT-B);

b Wender Utah Rating Scale – short version.

All individuals participated voluntarily in the study and gave written informed consent prior to neuropsychological assessment. Individuals were debriefed at the end of the assessment.

### Materials

#### 1. Standard measures of cognitive functions

The *Digit Span Forward task*, a subtest of the *Wechsler Memory Scale* [41], was applied as a measure of short-term memory. Series of numbers were read to the participants who were required to repeat the digits in the same order as presented. The number of correctly repeated sequences was registered.

The *Digit Span Backward task*, a subtest of the *Wechsler Memory Scale* [41], was applied as measure of working memory. Series of numbers were read to the participants who were required to repeat the digits in the reversed order. The number of correctly repeated sequences was registered.

Focused attention was measured with a computerized test (*Visual Scanning*) of the *Test Battery for Attentional Performance* [42]. In the *Visual Scanning task* a series of matrices (of about 8.8 by 8.8 cm) was presented in the center of the computer screen. Matrices were presented one at a time in consecutive order. Each matrix consisted of a regular array of 25 squares (each of about 1.2 by 1.2 cm, arranged in 5 lines and 5 columns). Each square had an opening on one of its sides (top, bottom, left or right side). A square with an opening at the top was defined as a critical stimulus throughout the task. The critical stimulus occurred only once in a matrix and was randomly distributed across the matrix. For each matrix presented, participants were asked to identify as quickly as possibly whether the matrix contained a critical stimulus or not. Participants responded by pressing the left button (if a matrix contained the critical stimulus) or the right button (if a matrix did not contain the critical stimulus). Immediately after a response (button press), a subsequent matrix was presented. A total of 50 trials were presented (25 matrices with the critical stimulus and 25 matrices without the critical stimulus). The sequence of trials was random. The reaction time for correct responses was registered.

Cognitive flexibility was measured with a computerized test (*Flexibility*) of the *Test Battery for Attentional Performance* [42]. The ﬂexibility task required the participants to place each hand on a separate response button, one button on the left and the other button on the right side, while viewing a computer screen. On the screen, a letter and a digit number (of about 12 by 16 mm) were displayed simultaneously, one on each side of the screen. The distance between the letter and the digit number was 5 cm. Participants were instructed to respond by alternately pressing the button that was on the same side of the screen as the letter, and then pressing the button that was on the same side of the screen as the number. This means that participants responded alternatingly to letter, number, letter, number, etc. throughout the task. After each response, a new letter and number appeared, randomly assigned to either side of the screen. The task started with a response to the letter. This means that the participants’ task was to respond to the letter by pressing the response button on the side of the letter (e.g. if the letter was presented on the right hand side of the center of the screen, participants were supposed to press the response button on the right side). Immediately after this trial, a new pair of stimuli (consisting of a new letter and a new number) was displayed. Participants were supposed to press the response button on that side on which the number was presented (right response button if the number was presented on the right hand side or left response button if the number was presented on the left hand side). Immediately after the response another pair of stimuli was presented and participants were again supposed to press the left or right response button according to whether the letter appeared to the left or the right hand side. A total of 100 trials were presented. The number of commission errors was calculated as a measure of flexibility.

Inhibition was measured with the *Stroop Color-Word Interference task* [43], [44]. The *Stroop Color-Word Interference task* consisted of three conditions. First, in the *Color Word* condition, color words (YELLOW, GREEN, BLUE and RED) printed in black ink were presented on a card and the participants were required to read them in clear voice as fast as possible. Second, in the *Color Block* condition, colored rectangles (rectangles printed in yellow, green, blue and red) were presented on a card and the participants were required to name the color of the rectangles as fast as possible. Third, in the *Color-Word Interference* condition, color words (YELLOW, GREEN, BLUE and RED) were presented and printed in mismatching ink (e.g. RED printed in blue ink). The participants were required to name the color of the words as fast as possible and to ignore the meaning of the printed word. Each trial consisted of the same number of stimuli. The time in seconds to complete each trial was registered. As dependent variable, a difference score was calculated per participant as a measure of inhibition by subtracting the time needed for completion of the *Color Block* condition from the *Color-Word Interference* condition [10].

Intellectual functions (IQ) were measured using the *Multiple Choice Vocabulary Test* [40]. This test consists of 37 lines, each comprising of one authentic word and four fictitious words. The participants were required to find the authentic word by underlining it. The Multiple Choice Vocabulary Test is a valid and short test procedure which provides a measure for intellectual functioning.

#### 2. Measurement of complex prospective memory

A planning task was developed based on the paradigm as devised by Kliegel and colleagues [33] in order to measure complex prospective memory. In the present task, participants were asked to plan and carry out 10 subtests in a limited period of time with the overall aim to maximize the total score. During the task, *task planning*, *plan recall*, *self-initiation, plan fidelity* and *task switching* were assessed.

In total, the following 10 subtests were designed which were grouped in five pairs of subtests (version A and B): Two subtests of arithmetic problems were designed (1A and 1B), each containing 68 mathematic equations (e.g. *3 × 8 =  ?*). The 68 equations were listed on a sheet in two columns. The participants were required to read and complete the mathematic equations verbally in clear voice. They were further instructed to begin at the upper left but were allowed to skip mathematical equations if necessary. A total of 136 equations were created. Equations were randomly allocated to one of the two subtests (1A and 1B) which contained 68 equations each. Two cancellation subtests (2a and 2B) were designed and were based on the *d2-Test of Attention* [45]. In each version of the subtest, a large number of visual stimuli were presented on a sheet. The items were arranged in 14 lines, each line containing 32 items. Predefined target stimuli within the large set of distractor stimuli had to be identified and crossed out with a pen. The cancellation tasks had to be performed systematically line by line, starting at the upper left. The two versions of the subtest (2A and 2B) consisted of the same stimuli, differently arranged on the sheet. Furthermore, two word finding subtests were designed (3A and 3B). In each version, a number of 102 incomplete words with some letters missing (e.g. *Ba_ketba_l* for *Basketball*) were presented on a sheet in three columns. The participants were required to complete the words in mind and to name them in clear voice (i.e. “Basketball”). The subtests were performed verbally and the participants were instructed to begin at the upper left, but were allowed to skip words if necessary. A total of 204 incomplete words were created. Incomplete words were randomly allocated to one of the two subtests (3A and 3B) which contained 102 incomplete words each. Moreover, two screwing subtests were included (4A and 4B). In each version of the screwing test, a nut needed to be moved on a screw by using both hands (length: 10.0 cm; diameter: 0.8 cm). The nuts were already placed on the screws. Direction and speed of the movement was not stipulated. The participants were instructed to keep a constant and regular speed. As the critical measure was the regularity of screwing, there was no predefined point when to stop working on the subtest. The two versions of the screwing subtests consisted of the same kind of screws, labeled with “A” and “B”. Finally, two ball squeezing subtests were included (5A and 5B). In each version, two foamed plastic balls (diameter: 5.5 cm) had to be squeezed and released repeatedly and simultaneously, one ball with each hand. Strength and speed of the movement was not stipulated. The participants were instructed to keep a constant and regular speed. As the critical measure was the regularity of ball squeezing, there was no predefined point when to stop working on the subtest. The two versions of the ball squeezing subtest consisted of the same kind of foamed plastic balls, labeled with “A” and “B”.

Similar to other planning tasks (e.g. The Six Elements Test [46]) or complex prospective memory tasks [33], the following rules were applied: Participants were requested to develop a plan for executing all subtests in a way that the overall score is maximized. The rules of the present task included (I) a restriction in time for task performance, i.e. the total time to work on all subtests was limited to 10 minutes, (II) a restriction in the sequence in which the subtests can be performed, i.e. it was not allowed to execute subtests of the same kind (e.g. 1A and 1B) immediately after each other, (III) the possibility to perform two subtests simultaneously (so-called dual-task units, e.g. performing the verbal task 1A while performing the motor task 4A) in order to obtain extra points which are added to the overall score and (IV) the instruction to work on each of the 10 subtests at least once for a short period of time. All subtests were designed in a way that the participants were not able to complete any subtest within five minutes (there was no predefined endpoint for subtests 4 and subtests 5). Therefore, participants were required to consciously switch between subtests in order to work on each subtest for at least a short period of time within the total duration of 10 minutes. Moreover, the subtests were designed to allow for dual-task units in a distinct way: While verbal arithmetic problems (subtests 1) and the verbal word finding tasks (subtests 3) could be performed simultaneously with motor tasks (subtests 4 and subtests 5), the written cancellation tasks (subtests 2) could not be combined with motor tasks. The reason to include these motor tasks was to increase complexity to the present paradigm (e.g. additional rules had to be considered) and thereby to increase demands in task planning, storage, recall and execution (i.e. through so-called dual-task units).

During the task, four components of complex prospective memory were assessed, including *task planning*, *plan recall*, *self-initiation* and *plan fidelity.* Since successful execution of the paradigm of complex prospective memory requires active switching between subtests, and because switching between subtests represents an action which is above the subtest level, task switching is crucial in the paradigm applied and may indicate general task performance (task switching). The aim of the present paradigm was not to assess the specific abilities as measured by an individual subtest (e.g., mathematical skills as indicated by the number of correctly solved mathematical problems), but to examine planning and delayed plan execution. Therefore, the performance within the subtests on the single items was not taken into consideration (e.g. number of solved mathematical equations) (see [29], [34]). Details of the assessment of the components of complex prospective memory are outlined below in the procedure section. In *task planning*, the participants were requested to formulate a precise plan for executing the subtests by following the rules as outlined above. The plan quality was scored based on a scoring scheme. Points were awarded for several aspects, including one point for each subtest to be initiated, one point for each time the version of the subtest (version A or version B) was specified, one point for each justification of a specific order (e.g. “I start with 1A because I am very good in mathematics”), one point for each time the duration intended to work on an individual subtest was specified, one point for each rule explicitly mentioned and one point for each planned dual-task unit. Two points are deducted for each rule violation. A sum score was calculated as a measure of plan quality. The maximum score was in principle unlimited (a similar procedure is outlined by Kliegel and colleauges [33]). *Plan recall* (retention) was assessed after a delay of about 40 minutes. The participants were requested to verbally recall the plan as precisely as possible. Plan recall was measured by the percentage of recalled subtests in the correct order based on the initial plan. In task *self-initiation*, the participants were instructed to self-initiate task execution at a predefined event (see below). It was noted by the experimenter if the participants succeeded to self-initiate task execution at the appropriate moment or if they forgot to do so. A measure of *plan fidelity* was obtained by calculating the percentage of the number of actually executed subtests in the correct order according to the original plan. A measure of *task switching* indicated general task performance. The number of actually initiated subtests were calculated and added to the number of executed dual-task units. The number of rule violations (e.g. 1A directly performed after 1B) was deducted from the score.

### Procedure

All participants were tested individually. At the beginning of the experiment, the participant gave written informed consent to participate in the study. Subsequently, the task of complex prospective memory was conducted. The test procedure consisted of three stages, (1) *introduction and planning*, (2) *retention and recalling* and (3) *initiation and execution*.

#### 1. Introduction and task planning

Test materials were presented to the participants and subtests were introduced. The participants were given the possibility to ask questions with regard to the subtests and the experimenter ensured comprehension of participants by asking questions about the subtests. Subsequently, the rules were outlined. On request, rules were repeatedly outlined. The participants were asked further questions to ensure rule comprehension. All participants were able to answer these questions so that no participant had to be excluded. Subsequently, the participants were asked to develop a plan how to execute the different subtests. The plan had to be described verbally and was digitally recorded for a later scoring of *task planning*. Participants were asked to report their developed plan as precisely and in as much detail as possible. They were further pointed to the digital recording of the description of their plan for a later scoring of their planning ability. Moreover, the participants were not allowed to take notes and the plan had to be retained in memory. Once a plan has been created and stored on a voice recorder, the participants were instructed that the plan had to be executed at a later time of the assessment. The test materials were stored in a box and placed to the right hand side of the participant under the table (out of sight of the participants). The participants were required to self-initiate the execution of their individual plan at the time when they will be asked for their age. The participants were informed to stop any ongoing tasks at this particular time, to reach for the box with the test materials and to start executing the tasks as previously planned. It was emphasized to the participants that no further reminder will be given except of the question about the age. The participants were further instructed to stick with the initial plan as close as possible. In case of uncertainties about the initial plan, the overall goal of maximizing the total score should be followed.

#### 2. Retention and recalling

A delay for about 60 minutes followed in which distractor tasks were performed. During this time period, other cognitive functions were assessed including measures of short-term memory, working memory, focused attention, flexibility, inhibition and intellectual functions. Within this period, after about 40 minutes, the participants were required to recall the initial plan for the prospective memory paradigm which was digitally recorded for a later scoring of *plan recall*. Retrospective memory of plan information was assessed after a rather long delay of about 40 minutes in order to create high demands on retention of plan information. After retrospective memory functions had been assessed, only a shorter time delay followed before plan execution was examined in order to reduce the confounding of measures by additional demanding retrospective memory requirements.

#### 3. Initializing and execution

After the 60 minutes delay (distractor tasks and plan recall), the participants were handed over a form asking for descriptive information such as age, gender and place of residence. When being asked for age, the participants were supposed to stop completing the form and to immediately start working on the prospective memory task by their own initiative. If participants failed to do so, they were given the time to complete the form. After this, the participants were immediately prompted to reach for the box with the test materials for the prospective memory task and to start executing the task. The participants were reminded to stick to their initial plan as close as possible. It was noted whether the participants successfully self-initiated the task (measure of *self-initiation*). A stop watch was placed next to the participants to give them the possibility to keep track of time. The sequence of executed subtests was noted by the experimenter for a later scoring of *plan fidelity* and *task switching*.

All participants were debriefed at the end of the assessment. The total duration of the assessment was about 90 minutes.

### Ethics Statement

The study was conducted in compliance with the Helsinki Declaration. Ethical approval was obtained by the ethics committee of the medical faculty of the University of Heidelberg, Germany. All participants gave written informed consent prior to the assessment.

### Statistical Analysis

Complex prospective memory performance and other cognitive functions were compared between patients with ADHD and healthy individuals using multivariate analysis of variance (MANOVA) and nonparametric statistical tests (χ^2^-test) for nominal data. Effect sizes (η^2^, Cohen’s d, Cohen’s ω) were calculated for all comparisons. As described by Cohen [36], η^2^ is a function of the effect size index f. According to Cohen [36], a small effect size (f = .10) corresponds to an η^2^ = .0099, a medium effect size (f = .25) to an η^2^ = .0588 and a large effect size (f = .40) to an η^2^ = .1379. For pairwise comparisons of means, negligible effects (d <0.20), small effects (d = 0.20), medium effects (d = 0.50) and large effects (d = 0.80) were distinguished [36]. To interpret effect sizes for χ^2^-test for nominal data, negligible effects (ω <0.1), small effects (ω = 0.1), medium effects (ω = 0.3) and large effects (ω = 0.5) were distinguished [36]. Furthermore, Pearson product-moment correlations were applied exploratory to test for significant relationships between components of prospective memory, separately for the group of patients with ADHD and healthy individuals. With respect to correlation analysis, negligible effects (r <0.1), small effects (r = 0.1), medium effects (r = 0.3) and large effects (r = 0.5) were distinguished [36]. To determine which cognitive functions (i.e. short-term memory, working memory, focused attention, flexibility and inhibition) contribute to performances on complex prospective memory, multiple regression analyses were performed. Regression analyses were carried out separately on patients with ADHD and healthy individuals. A stepwise multiple regression analysis (inclusion of predictor variables in a forward selection method) was calculated for those components of prospective memory which were found to differ significantly between groups. A significance level of α = .05 was initially applied on all tests. However, complex prospective memory performance was compared between patients and healthy individuals on five variables which results in α-error accumulation. Therefore, the significance level α was adjusted by using a Bonferroni correction to control for the problem of multiple comparisons. Adjustment of significance level was not applied on correlation analyses and also not on the comparison between groups with regard to cognitive functioning (e.g. short-term memory and working memory, etc.) as assessed by standard tests of cognition. Those analyses were carried out on an exploratory level and interpretations are primarily based on effect sizes. Data analysis was performed using SPSS 18 for Windows.

## Results

### Performance in Complex Prospective Memory

Multiple analysis of variance (MANOVA) indicated a large and significant difference between patients with ADHD and healthy individuals (Wilk’s lambda = 0.585, F(4,85) = 15.06, p<.001, η^2^ = .415) with regard to complex prospective memory. In comparison to healthy individuals, patients with ADHD created less elaborate plans (*task planning*) (F(1,88) = 56.03, p<.001) and displayed more difficulties in *task switching* (F(1,88) = 19.42, p<.001). These effects were of large size (task planning: d = 1.60, task switching: d = 0.94). No significant differences were observed in *plan recall* (F(1,88) = 0.02 p = .878), *self-initiation* (χ^2^ (1) = 2.18; p = .140) and *plan fidelity* (F(1,88) = 3.98, p = .049). A Bonferroni corrected significance level of α = .01 was applied. Test performances of groups and effect sizes are presented in Table 2.

**Table 2:** Performance in complex prospective memory.

	Patients with ADHD (n = 45)	Healthy individuals (n = 45)	Effect size^f^
Task planning^a^	11.6±5.8	20.3±5.2	d = 1.60*
Plan recall^b^	86.5±20.5	86.7±20.3	d = 0.03
Self-initiation^c^	18/27	25/20	ω = 0.16
Plan fidelity^d^	59.8±26.8	70.3±23.2	d = 0.43
Task switching^e^	6.4±4.2	11.2±5.8	d = 0.94*

a Planning score;

b Percentage of recalled subtests in the correct order according initial plan;

c Number of participants who did self-initiate/did not initiate plan execution;

d Percentage of executed subtests in the correct order according to initial plan;

e Switching score;

f Effect sizes indicated by Cohen’s d or Cohen’s ω;

* Significant at p<.01.

### Relationship between Different Components of Complex Prospective Memory

Table 3 presents Pearson product-moment correlation coefficients between components of prospective memory individually for the group of patients and healthy individuals. Exploratory data analysis revealed no significant relationship between task planning, plan recall, self-initiation and plan fidelity for both groups with the exception of the relationship between plan recall and plan fidelity for the control group (r = 0.39; p = .008). Regarding task switching as a general indicator of task performance, significant correlations were found between task switching and task planning (r = 0.35; p = .018) and between task switching and plan fidelity (r = 0.42; p = .004) for the control group. The group of patients showed a significant correlation between task switching and self-initiation of task execution (r = 0.37; p = .011) and between task switching and plan fidelity (r = 0.56, p<.001). All significant correlations were of medium size except for the relationship between task switching and plan fidelity of patients which was of large size.

**Table 3:** Pearson product-moment correlations between components of prospective memory for patients with ADHD (r_ADHD_) and healthy individuals as control participants (r_c_).

	Plan recall	Self-initiation	Plan fidelity	Task switching
Task planning	r_ADHD_ = −.08	r_ADHD_ = .09	r_ADHD_ = −.06	r_ADHD_ = .26
	p_ADHD_ = .611	p_ADHD_ = .548	p_ADHD_ = .702	p_ADHD_ = .083
	r_c_ = .10	r_c_ = .11	r_c_ = .11	r_c_ = .35
	p_c_ = .512	p_c_ = .483	p_c_ = .468	p_c_ = .018*
Plan recall	–	r_ADHD_ = .24	r_ADHD_ = .24	r_ADHD_ = −.03
		p_ADHD_ = .116	p_ADHD_ = .116	p_ADHD_ = .846
		r_c_ = .07	r_c_ = .39	r_c_ = .19
		p_c_ = .632	p_c_ = .008*	p_c_ = .219
Self-initiation		–	r_ADHD_ = .26	r_ADHD_ = .37
			p_ADHD_ = .090	p_ADHD_ = .011*
			r_c_ = .03	r_c_ = .07
			p_c_ = .852	p_c_ = .645
Plan fidelity			–	r_ADHD_ = .56
				p_ADHD_ <.001*
				r_c_ = .42
				p_c_ = .004*

* Significant at p<.05.

### Performance in Standard Tests of Cognitive Functions

Comparison between patients’ and healthy individuals’ cognitive functioning using MANOVA revealed a large-scale difference (MANOVA: Wilk’s lambda = 0.713, F(5,82) = 6.597, p<.001, η^2^ = .287). Subsequent analyses showed that patients displayed a decreased performance in short-term memory (F(1,86) = 4.05, p = .047), focused attention (F(1,86) = 9.04, p = .003), flexibility (F(1,86) = 5.54, p = .021) and inhibition (F(1,86) = 24.73, p<.001). No group difference was observed for working memory performance (F(1,86) = 1.41, p = .24). The effect sizes between groups ranged from small to large effects (Table 4).

**Table 4:** Standard measures of cognitive functions for patients with ADHD and healthy individuals.

	Patients with ADHD (n = 45)	Healthy individuals (n = 45)	Effect size f
Short-term memory^a^	6.9±1.8	7.7±2.2	d = 0.40*
Working memory^b^	6.3±1.9	6.7±2.2	d = 0.20
Focused attention^c^	5.5±1.6	4.5±1.4	d = 0.67*
Flexibility^d^	3.9±4.7	1.8±2.8	d = 0.55*
Inhibition^e^	39.5±16.3	25.3±9.8	d = 1.07*

a Digit Span Forward task;

b Digit Span Backward task;

c Visual Scanning (TAP);

d Flexibility (TAP);

e Stroop Color-Word Interference task;

f Effect size indicated by Cohen’s d

* Significant at p<.05.

Furthermore, the contribution of standard measures of cognitive functions on components of complex prospective memory (*task planning* and *task switching*) was explored by multiple regression analyses. Regarding patients with ADHD, a significant model to predict *task planning* was found in a stepwise selection method of predictor variables (forward selection method; F(1,44) = 6.43, p = .015). Among all standard measures of cognition, only inhibition contributed significantly to *task planning* which explained 13.3% of the total variance (Table 5). No model was obtained to predict *task switching* in patients with ADHD (no variables entered the model on α = .05). Regarding healthy individuals, no significant model was found to predict either *task planning* or *task switching* (α = .05).

**Table 5:** Summary of multiple regression analysis (stepwise inclusion of predictors in a forward selection method) of standard measures of cognition on task planning for adults with ADHD.

Predictor variables	B	SE B	β	t	p
*Model*					
Inhibition^a^	−0.13	0.05	−0.36	−2.54	.015*
Total R^2^ = 13.3*					
*Excluded Variables*					
Short-term memory^b^				0.66	.51
Working memory^c^				−1.52	.14
Focused attention^d^				−0.27	.79
Flexibility^e^				0.53	.60

a Stroop Color-Word Interference task;

b Digit Span Forward task;

c Digit Span Backward task;

d Visual Scanning (TAP);

e Flexibility (TAP);

* Significant at p<.05.

## Discussion

Prospective memory is crucial for everyday occupational and social functioning. In the present study, complex prospective memory was explored in adult patients with ADHD which was described as a realistic approach in the assessment of prospective memory [33]. Successful functioning in prospective memory requires the completion of several subtasks, making an individual examination of the involved cognitive components necessary. As hypothesized, a large-scale deficit in task planning was observed for patients with ADHD. In comparison to healthy participants, the group of patients showed impairments in the formation of multiple intentions and hence created less elaborate plans. In this respect, the present results are in accordance with previous research on complex prospective memory in patients with ADHD, which found inefficient planning abilities in both children and adults with ADHD [29], [31]. The findings in children with ADHD as reported by Kliegel and colleagues [29] however cannot be fully applied, since only a small subgroup of healthy children (8 of 20 children) but all children with ADHD (20 children) made an explicit plan on how to work on the task. In the present study, both groups recalled their individual plans with high accuracy after a delay of 40 minutes (86.5% for patients, 86.7% for healthy individuals). Even though patients created less elaborate plans, data analysis revealed that they succeeded to encode, store and retrieve plan information. Thus, retrospective memory requirements in complex task planning can be assumed to be intact in patients with ADHD. The repeatedly demonstrated impairments in verbal memory functions in adults with ADHD [13], [14] therefore appear not to affect encoding and retrieval of multiple intentions even if a complex plan containing several subtasks has to be stored. Furthermore, the present study examined for the first time task self-initiation as a component of complex prospective memory in adults with ADHD. The ability to recall at a certain time or event in the future that an action or task has to be executed is the core of prospective memory. Consequently, self-initiation of behavior is crucial in prospective memory. However, self-initiation cannot be regarded as an isolated action as plans need to be (I) created, (II) stored in memory and (III) executed at a later time. Eighteen patients with ADHD (40%) and 25 healthy individuals (56%) were successful in self-initiation at the right event. This difference did not reach significance and is therefore in agreement with previous findings showing no impairments in event-based task self-initiation but indicating impairments in time-based task self-initiation in both children and adults with ADHD [27], [28], [30], [31]. Furthermore, findings are difficult to compare as the cue to signify the moment when to start with the task execution differ throughout paradigms [28], [29]. This is crucial since research showed that the saliency of the prospective cue has strong effects on the success of prospective remembering [47]. Finally, at task execution, patients with ADHD and healthy individuals did not differ considerably in plan adherence. The results in task execution are of particular interest as both groups have demonstrated successful plan encoding and plan recall before plan execution was assessed. In contrast, impaired plan adherence in adults with ADHD was demonstrated by Altgassen and colleagues [31], however, this study did not assess plan recall so that it remained unclear whether the plan was inadequately encoded in memory or whether deviation from the original plan appeared at the time of execution.

In general, the present study revealed that deficits in complex prospective memory of adults with ADHD mainly emerged from considerable impairments in task planning. Plan recall of multiple intentions, self-initiation and task execution appeared to be intact. Exploratory correlation analyses between components of complex prospective memory (planning, plan recall, self-initiation and plan fidelity) revealed no significant relationship between any of the components for the group of patients and only one significant positive relationship was observed for the control group (between plan recall and plan fidelity). It can be concluded that complex prospective memory consists of four cognitive components which are largely independent from each other. Data analysis showed a specific impairment in task planning of adults with ADHD suggesting that the observed impairment in prospective memory is not resulting from a global cognitive deficit but may rather reflect a very differential effect of impaired executive functioning. Since other components of prospective memory (i.e. self-initiation and plan fidelity) also require interrelated executive functions including attention, initiating actions or consistent monitoring of actions in relation to a defined plan or goal, the observed impairment is also not the consequence of a global but rather a very specific deficit of executive functioning, i.e. a planning deficit. This assumption is supported by the findings of studies reporting considerable impairments of adults with ADHD with regard to planning and problem solving [11], [48]. Despite various components of prospective memory were not found to be impaired in adults with ADHD (with the exception of task planning), patients showed a severe impairment in prospective memory as indicated by a reduced general task performance (task switching). Correlation analyses demonstrated significant relationships between task switching and task planning, self-initiation as well as plan fidelity in healthy individuals and/or patients, but no association with plan retention. This result supports the notion that task planning, self-initiation and plan fidelity all require interrelated executive functions (as reflected by correlations with task switching), whereas plan retention primarily relies on retrospective memory functions. Furthermore, in accordance with previous findings on ADHD [3], [6], [49], exploratory analysis of the present data showed impairments in patients with ADHD on standardized measures of short-term memory, focused attention, flexibility and inhibition.

Multiple regression analyses were performed on those components of prospective memory in which adults with ADHD were found to be impaired (task planning and task switching). With regard to patients with ADHD, regression analyses identified inhibition to contribute significantly to task planning by explaining a considerable amount of variance (13.3%), whereas no model was found to predict task switching. In healthy individuals, however, no significant model was found to predict either task planning or task switching. Results from regression analyses therefore suggest that inhibition serves as a predictor of impaired functioning in prospective memory which is consistent with evidence from previous studies on prospective memory which identified inhibition as an important mediator of planning deficits in patients with Parkinson’s disease [34]. Furthermore, the present results further confirm the findings of Altgassen and colleagues [31], who discussed differences in inhibitory load as a potential candidate to explain differential effects of time-based and event-based prospective memory capacities in individuals with ADHD. Inhibitory control can be hypothesized to link complex prospective memory requirements with time-based and event-based prospective memory functions. Nevertheless, a qualitative differentiation is supported by the findings of Altgassen and colleagues [31], who found mainly weak correlations between measures of time-based, event-based and complex prospective memory. In this respect, complex prospective memory can be distinguished from time-based and event-based prospective memory and might represent a different quality of prospective memory focusing on several cognitive processes involved, including planning, sequencing and execution of multiple delayed intentions.

However, the present study has to be regarded in the context of some limitations. The cue to self-initiate the execution of their individual plan was a particular event (i.e. a question in a questionnaire about personal descriptive information), whereby a time-based cue to self-initiate task execution was not included. Furthermore, participants were only given once the opportunity to self-initiate task execution within a defined, brief time window. The reliability of single, one-time prospective memory tasks can be questioned and therefore tasks requiring actions on multiple times/events should be preferred. Moreover, the present paradigm can be described as a short-term task of prospective memory as it is completed within a structured test session. In contrast, long-term tasks are performed hours or days after the actual assessment and are considered to be more naturalistic as they represent typical everyday prospective memory tasks [50]. Most current measurement tools for the assessment of prospective memory (e.g. the CAMPROMPT) are not restricted to a single time window but require participants to initiate tasks at multiple times/events and thereby mix time-based and event-based task requirements [51]–[54]. Moreover, some assessment tools (such as the MIST and the RPA-ProMem) include both short-term tasks (to be performed within an actual test session) and long-term tasks (to be performed outside the laboratory setting) to mirror naturalistic prospective memory tasks in everyday life [52]–[54]. Those measures of prospective memory have been shown to yield good reliability and validity and can be regarded as very useful assessment tools for both research and clinical application [53]–[56]. However, the strength of the present paradigm of complex prospective memory is the assessment of individual phases in prospective remembering. The developed task examines different cognitive components involved in prospective memory, such as planning, sequencing and coordinating of multiple intentions. The paradigm thereby allows an identification of particular deficits in unsuccessful prospective memory which may have relevant therapeutic implications for clinical practice. The core deficits of adult patients with ADHD as found in the present study were difficulties in elaborate task planning. Implications can be drawn for daily practice and for behavioral based intervention strategies such as cognitive-behavioral therapy (CBT). In clinical settings, agreements and intentions for behavioral changes are made between patients and clinicians that need to be implemented in patients’ daily life (e.g. structuring daily routines, keeping appropriate interactions with colleagues, controlling of impulsive behaviors, taking medication). Our results suggest that these intentions need to be carefully planned and prepared. Moreover, external help might be necessary for patients to achieve this (e.g. by a therapist, coach or family member). Once intentions are formed, patients with ADHD are able to store them in memory and, at the appropriate event, are able to self-initiate and execute them to the same accuracy as healthy individuals. In contrast to commonly assumed deficits, patients with ADHD are not unreliable in the realization of intentions if agreements have been well structured and organized in clinical settings. Therefore, clinicians are advised to focus on elaborate and careful planning of delayed intentions in order to induce reliable behavioral changes in the treatment of patients with ADHD.

Moreover, it would be of importance to gain knowledge about the effects of pharmacological treatment interventions (i.e. stimulant medication) on prospective memory performance in adults with ADHD. Previous research demonstrated the efficacy of stimulant medication on clinical outcome [57], [58] and cognitive functions such as memory, attention and problem solving [6], [11], [59]. However, it is still unclear whether stimulant drug treatment improves planning abilities in complex prospective memory and whether patients achieve a higher level of functioning in the execution of delayed intentions. Future research should address the issue of pharmacological interventions in complex prospective memory in general, and planning abilities and inhibitory control in particular.
